# CXCL8 Chemokines in Teleost Fish: Two Lineages with Distinct Expression Profiles during Early Phases of Inflammation

**DOI:** 10.1371/journal.pone.0012384

**Published:** 2010-08-26

**Authors:** Lieke M. van der Aa, Magdalena Chadzinska, Edwin Tijhaar, Pierre Boudinot, B. M. Lidy Verburg-van Kemenade

**Affiliations:** 1 Cell Biology and Immunology Group, Department of Animal Sciences, Wageningen University, Wageningen, The Netherlands; 2 Virologie et Immunologie Moléculaires, Institut National de la Recherche Agronomique, Jouy-en-Josas, France; 3 Department of Evolutionary Immunobiology, Institute of Zoology, Jagiellonian University, Krakow, Poland; University of California Los Angeles, United States of America

## Abstract

**Background:**

During the inflammatory process, chemokine CXCL8 plays a pivotal role in recruitment of human neutrophilic granulocytes. A diversity of sequences similar to CXCL8 was reported in fish, but their evolutionary relationships and functional homology with their human homolog remain unclear.

**Principal Findings:**

We screened fish genomes to seek for sequences related to CXCL8. A first lineage was retrieved in all teleosts, while a second CXCL8 lineage was found in zebrafish and carp only. An early inflammatory function for both lineages was indicated by several lines of evidence. The induction of carp CXCL8s, CXCb, and CXC receptor-1 and -2 was analyzed after *in vitro* stimulation of leukocyte subpopulations and in two *in vivo* inflammation models. Recombinant proteins of carp CXCL8 proteins were produced and showed significant chemotactic activity for carp leukocytes.

**Conclusions:**

While both carp CXCL8s appear to be functional homologs of mammalian CXCL8, their different induction requirements and kinetics evoke a gene-specific sub-functionalization.

## Introduction

Chemokines are specialized cytokines with chemotactic activity and orchestrate mobilization and migration of specific subsets of cells along a gradient. Although initially discovered to be involved in leukocyte recruitment during early inflammation, they are now known to regulate various steps of the immune response, and to direct cell migration during growth and development. Moreover, chemokines are implicated in cancer by regulating the development of metastasis (reviewed by [1]). CXCL8, also known as IL-8 for interleukin-8, or NAP-1 for neutrophil-activating peptide, was the first chemokine discovered. It was initially purified from LPS-stimulated human blood monocytes [2], [3] and it is now recognized that CXCL8 is produced by a wide range of cell types including non-immune cells, like fibroblasts and endothelial cells [4], [5]. Besides its potent chemotactic activity for neutrophils, basophils, resting T cells, and stimulated eosinophils, CXCL8 also activates cells by induction of respiratory burst, exocytosis and degranulation of storage proteins and production of lipid mediators [6], [7], [8]. Moreover, CXCL8 regulates growth of endothelial cells and myeloid progenitor division [9], [10].

CXCL8 is classified into the CXC subfamily, based on the presence of the CXC cysteine-motif at the N-terminus. In human, sixteen CXC ligands are identified, of which the majority is located in mini-clusters on chromosome four [11], [12]. The human CXC proteins CXCL1, -2, -3, -5, -6, -7 and -8 contain an ELR (Glu-Leu-Arg) signature upstream of the CXC motif which is important for receptor affinity. In general, human CXC chemokines with the ELR signature recruit polymorphonuclear leukocytes (PMN, e.g. neutrophils, basophils and eosinophils) during inflammation and promote angiogenesis. In contrast, CXC chemokines lacking the ELR signature specifically attract lymphocytes and monocytes, not neutrophils, and they inhibit angiogenesis [13]. The receptors for CXCL8 in humans, CXCR1 and CXCR2, are promiscuous and also bind other chemokines including CXCL1, -4 and -7 [14], [15]. Both receptors are highly expressed on human neutrophils [16], but CXCR1 is also well expressed on many other cell types.

Orthologs for human CXCL8 are identified in other mammals as monkeys, cow, dog, cat, but not in mouse and rat. Outside mammals, CXCL8-related genes have been described in chicken [17], [18] and in multiple teleost fish [19], [20], [21], [22], [23], [24], [25], [26], [27]. Orthology of fish CXCL8-related sequences with mammalian CXCL8 has currently not been firmly shown. The chemokine family of ligands and receptors has evolved in a distinctive way in fish, compared to tetrapods [12], [18], [26], [28]. For example, the CC chemokine family has expanded and diversified extensively in zebrafish and in total 111 chemokine genes have been identified in the zebrafish genome, against 44 genes in humans [26]. Moreover, a fifth chemokine subgroup with a CX signature exists in zebrafish [26]. While some of the mammalian CXC ligands have unambiguous orthologs in fish, such as CXCL12 and CXCL14 [29], other CXC genes form fish-specific lineages [26], [28], [30]. Since these proteins are short and evolve quickly, it is often difficult to show more than a tendency in distance by phylogenetic analysis, especially when only a few sequences are available. Subsequently, CXCa was proposed as an alternative name for fish CXCL8-like genes, together with CXCb that designated chemokines from the fish lineage most similar, but probably not orthologous, to human CXCL9, -10 and -11 [21].

A specific feature of teleost CXCL8-related proteins is the absence of a conserved ELR signature, with the exception of haddock [22]. Nevertheless, first studies with recombinant proteins demonstrate a chemotactic activity for neutrophils and macrophages [25], [31], [32]. Furthermore, gene expression studies indicate that fish CXCL8s are pro-inflammatory cytokines, suggesting that they may fulfill similar functions in inflammation as mammalian CXCL8 [24], [33], [34].

Recently, a second CXCL8-related sequence was identified from a carp EST database that was named CaIL-8 [35]. CaIL-8 only shares low similarity to the previously described carp CXCa sequence, indicating that CaIL-8 is a second CXCL8-like gene. Moreover, CaIL-8 is slightly more similar to the human CXCL8 than to the carp CXCa [21]. Together with two other carp sequences and a zebrafish EST, the CaIL8 therefore constitutes a second fish CXCL8-lineage (CXCL8_L2). The earlier described teleost CXCL8-like genes, including CXCa of carp, form the first lineage and will now be referred to as CXCL8_L1.

In this study we further mined available databases for CXCL8_L1 and CXCL8_L2-like sequences in teleost fish and studied their phylogenetic relationships and synteny groups. To establish the functions of different carp chemokines during inflammation, we performed an extensive analysis of expression profiles of carp CXCL8_L2, CXCa_L1, CXCb and the receptors CXCR1 and CXCR2 after *in vitro* stimulation of leukocyte subpopulations. We moreover looked at selective expression of carp chemokines and receptors in two *in vivo* models of inflammation: a zymosan-induced peritonitis model and a model of hyperosmotic shock by immersion vaccination to *Aeromonas salmonicida*. Finally, we established that both the CXCL8_L1 and CXCL8_L2 chemokines truly represent functional chemotactic peptides using recombinant proteins.

## Methods

### 1. Bioinformatics, phylogeny and synteny

Carp CXCL8_L2 (GenBank accession number AB470924) and zebrafish CXCL8_L1_chr1 (GenBank accession number XM_001342570) sequences were used as query in BLAST searches. CXCL8_L1 genomic sequences were retrieved for zebrafish (*Danio rerio*), medaka (*Oryzias latipes*), tetraodon (*Tetraodon nigroviridis*), stickleback (*Gasterosteus aculeatus*) and fugu (*Takifugu rubripes*) from the EMSEMBL website (http://www.ensembl.org/index.html). CXCL8_L1 genes corresponded with Ensembl gene identifiers ENSTNIG00000017810 for tetraodon, ENSGACG00000001729 for stickleback, ENSORLG00000005096 for medaka and fugu Genbank accession number AB125645.1. BLAST searches against EST databases were performed on the website of NCBI (http://www.ncbi.nlm.nih.gov/). Zebrafish EST sequences corresponding to CXCL8_L2_chr17 were EH557944, EH536693, EH441857 and EH977746. Multiple sequence alignments were made with ClustalW within the *MEGA*4 software. Phylogeny trees were constructed with MEGA4 using Neighbour Joining (NJ; [36]) and Maximum Likelihood (ML; [37]). The online tool *Genomicus* (http://www.dyogen.ens.fr/genomicus-56.02/cgi-bin/search.pl) was employed to study conserved synteny for fish CXCL8 genes. *Genomicus* allows analysis of genes with approved gene symbols published on the ENSEMBL website. Since zebrafish CXCL8 genes are not yet annotated by ENSEMBL, annotated genes in close proximity to the genes of interest were used as reference genes in the analysis. *Camk2d2* was taken as a reference gene for zebrafish CXCL8_L1_chr1, *asb7* was used as a reference for zebrafish CXCL8_L2_chr7 and *C14orf104/kintoun* was used as a reference for zebrafish CXCL8_Chr17.

### 2. Animals

Young individuals (6–9 months) of common carp *Cyprinus carpio* L (50–60 g b.w), from the Department of Immunology, Polish Academy of Science, Golysz, Poland (R7xW) and “De Haar Vissen” facility in Wageningen (R3xR8) were reared at 23°C in recirculating tap water [38]. Fish were fed pelleted dry food (Trouvit, Nutreco). All animals were handled in strict accordance with good animal practice as defined by the relevant national and/or local animal welfare bodies, and all animal work was approved by the appropriate committee (license numbers: 3019b/2003 and 013b/2010, Wageningen University ethical committee; 16/OP/2001, Jagiellonian University ethical committee).

### 3. Tissue and section preparation

Fish were anaesthetized with 0.2 g/l tricaine methane sulphonate (TMS, Cresent Research Chemicals, Phoenix, AZ, USA) buffered with 0.4 g/l NaHCO_3_ (Merck, Darmstadt, Germany). Organs and tissues for RNA extraction were carefully removed, snap frozen in solid CO_2_ or liquid N_2_ and stored at −80°C.

### 4. *In vivo* study

#### 4.1. Hyperosmotic shock experiment

Fish were immersed in 4.5% (w/v) NaCl (1450 mOsm/kg aerated overnight before use) for 2 min and immediately net transferred to vaccine solution (LPS–DTAF (0.2% (w/v)), *A. salmonicida* bacterin-FITC (2.4×10^7^ bacteria/ml) or BSA-FITC (2% (w/v)) for 10 min (HI, hyperosmotic immersion, fish). The high salinity of the hyperosmotic solution caused the fish to passively float to the surface. DI (direct immersion) vaccinated fish were immersed in vaccine solution only for 10 min. Control fish were not exposed to NaCl and vaccin immersion. After vaccination fish were returned to their tanks. At selected time points, animals were sacrificed and their gills were isolated by carefully excising whole gill arches [39].

#### 4.2. Zymosan-induced peritonitis

The animals were i.p. injected with freshly prepared zymosan A (2 mg/ml, 1 ml/50 g b.w., Sigma, Z) in sterile PBS (270 mOsM) or with sterile PBS only (control group). At selected time points animals were sacrificed and their peritoneal cavities were lavaged with 1 ml of ice- cold PBS. Peritoneal leukocytes were centrifuged for 10 min at 800 g at 4°C and frozen in liquid nitrogen and stored at −80°C [33].

### 5. Cell isolation and *in vitro* culture

Animals were anaesthetized with 0.2 g/l TMS. Fish were bled through puncture of the caudal vein using a heparinized syringe.

#### 5.1. Isolation of peripheral blood leukocytes

Blood was centrifuged 5 min at 100 g and afterwards 10 min at 800 g and 4°C. The buffy coat and a small amount of serum were mixed and loaded on 3 ml Ficoll (density 1.077g/ml, Amersham Bioscience, Uppsala, Sweden). Following subsequent centrifugation at 800 g at 4°C for 25 min with the break disengaged, leukocytes at the interface were collected and washed twice with carp RPMI medium (cRPMI) (RPMI 1640, Invitrogen, Carlsbad, CA); adjusted to carp osmolarity (270 mOsm/kg) and containing 10 IU/ml heparin (Leo Pharmaceutical Products Ltd., Weesp, The Netherlands) and once with cRPMI++ (cRPMI supplemented with 0.5% (v/v) pooled carp serum, 1% L-glutamine (Merck, Whitehouse Station, NJ), 200 nM 2-mercaptoethanol (Bio-Rad, Hercules, CA), 1% (v/v) penicillin G (Sigma–Aldrich, St. Louis, MO), and 1% (v/v) streptomycin sulphate (Sigma–Aldrich, St. Louis, MO).

#### 5.2. Isolation of head kidney leukocytes

Head kidney cell suspensions were obtained by passing the tissue through a 50 µm nylon mesh with cRPMI and washed once. This cell suspension was layered on a discontinuous Percoll (Amersham Biosciences, Piscataway, NJ) gradient (1.02, 1.060, 1.070, and 1.083 g/cm^3^) and centrifuged for 30 min at 800 g with the brake disengaged.

#### 5.2.1. Isolation of head kidney monocytes/lymphocytes and phagocyte populations

Cells from the density range of 1.020–1.060 g/cm^3^ (predominantly, >80% monocytes/lymphocytes), the range of 1.060–1.070 g/cm^3^ (predominantly macrophages, but also monocytes, lymphocytes and some (∼10%) granulocytes), the range from 1.070 to 1.083 g/cm^3^ (∼80% neutrophilic granulocytes) [40] or combined 1.060–1.083 g/cm^3^ fractions (enriched phagocytes) were collected, washed, and seeded at 5×10^6^ cells per well (in a volume of 900 µl) in a 24-well cell culture plate at 27°C, 5% CO_2_ in cRPMI++.

#### 5.3. *In vitro* stimulation of cells

Cells were incubated 1–12 h with lipopolysaccharide (LPS, 10–50 µg/ml, *E. coli* serotype O55: B5, Sigma–Aldrich, St. Louis, MO), or with poly-inosinic poly cytidiylic acid (Poly I∶C, 50 µg/ml, Sigma–Aldrich, St. Louis, MO), phytohemagglutinin (PHA, 10 µg/ml, Sigma–Aldrich, St. Louis, MO). Control cells (C) received medium only.

### 6. RNA isolation and first strand cDNA synthesis

RNA was isolated using an RNeasy Mini Kit (Qiagen, Valencia, CA) according the manufacturer's protocol. Final elution was carried out in 30 µl of nuclease-free water, to maximize the concentration of RNA. RNA concentrations were measured by spectrophotometry and integrity was ensured by analysis on a 1% agarose gel before proceeding with cDNA synthesis.

For each sample a non–RT (non-reverse transcriptase) control was included. Two µl of 10× DNase I reaction buffer and 2 µl DNase I (Invitrogen) was added to 2 µg total RNA and incubated for 15 min at room temperature. DNase I was inactivated with 25mM EDTA (2 µl, 65°C, 10 min). To each sample, 2 µl random primers and 2 µl 10mM dNTP mix were added, and the mix was incubated for 5 min at 65°C and then 1 min on ice. After incubation, to each sample 8 µl 5× First Strand buffer 2 µl 0.1 M dithiothreitol (DTT) and 2 µl RNase inhibitor were added. To 19 µl from each sample (but not to the non-RT controls) 1 µl Superscript RNase H-Reverse Transcriptase (RT, Invitrogen) was added and reagents were incubated for 5 min at 25°C, then spun briefly and incubated 60 min at 50°C. Reactions were inactivated 15 min at 70°C. Samples were set at 100 µl with demineralized water and stored at −20°C until future used.

#### 6.1. Real-time quantitative PCR

PRIMER EXPRESS software (Applied Biosystems) was used to design primers for use in real-time quantitative PCR. Carp-specific primers (5′ to 3′) for chemokines: CXCL8_L2, CXCa_L1, CXCb and for chemokine receptors: CXCR1 and CXCR2 were used. The 40S ribosomal protein s11 gene served as an internal standard (accession numbers and primer sequences are listed in Table 1).

**Table 1:** Primers used for gene expression studies.

Gene	Sense (5′-3′)	Antisense (5′-3′)	acc. no
40S	CCGTGGGTGACATCGTTACA	TCAGGACATTGAACCTCACTGTCT	**AB012087**
CXCL8_L2	TCACTTCACTGGTGTTGCTC	GGAATTGCTGGCTCTGAATG	**AB470924**
CXCa_L1	CTGGGATTCCTGACCATTGGT	GTTGGCTCTCTGTTTCAATGCA	**AJ421443**
CXCb	GGGCAGGTGTTTTTGTGTTGA	AAGAGCGACTTGCGGGTATG	**AB082985**
CXCR1	GCAAATTGGTTAGCCTGGTGA	AGGCGACTCCACTGCACAA	**AB010468**
CXCR2	TATGTGCAAACTGATTTCAGGCTTAC	GCACACACTATACCAACCAGATGG	**AB010713**

For RQ-PCR 5 µl cDNA and forward and reverse primers (4.2 µM each) were added to 7 µl Absolute QPCR SYBR Green Mixes (ABgene). RQ-PCR (15 min at 95°C, 40 cycles of 15 s at 94°C, 30 s at 60°C, and 30 s at 72°C, followed by 1 min at 60°C) was carried out with a Rotorgene 2000 realtime cycler (Corbett Research, Sydney, Australia). Following each run, melt curves were collected by detecting fluorescence from 60 to 90°C at 1°C intervals.

Constitutive expression of chemokines was determined in various organs and tissues of four individual adult carp, and rendered as a ratio of target gene vs. reference gene (40S ribosomal protein s11 gene) calculated with the Pfaffl method [41], according to the following equation:
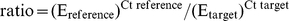
where E is the amplification efficiency and Ct is the number of PCR cycles needed for the signal to exceed a predetermined threshold value.

Expression following stimulation was rendered as a ratio of target gene vs. reference gene (40S ribosomal protein s11 gene) relative to expression in unstimulated control samples according to the following equation:
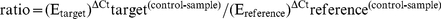



### 7. Cloning carp CXCa_L1 and carp CXCL8_L2

Synthetic genes encoding the mature carp CXCa_L1 (Genbank accession no. AJ550164) and carp CXCL8_L2 (Genbank accession no. AB470924), codon optimized for *E. coli* expression, were ordered at Mr. Gene (Regensburg, Germany). Restriction sites for *BamHI* and *HindIII* were included at respectively the 5′and 3′end of the coding sequences to enable cloning in the corresponding restriction sites of expression vector pET15new. This vector is a derivative from the vector pET15 (Novagen), that encodes for a N terminal tag containing 6 histidine residues under the control of a Lac operon and T7 promotor. The protein sequence for recombinant carp CXCa_L1 was:

MSYYHHHHHHLESGSMSLRGLGVDPR**C**R**C**IETESQRIGKLIESVELFPPSPH**C**KDTEIIATLKVSRKEI**C**LDPTAPWVKKVIEKIIANKTPAA

The protein sequence for recombinant carp CXCL8_L2 was:

MSYYHHHHHHLESGSRPKSQQLS**C**R**C**PRMHSEPAIPANKVLSLRVIPAGPI**C**KNENIIATMKKGQV**C**LDPTKDWVISLNEEIKKRNLKSQP, whereby the 6 histidine tag and LESGS epitope are underlined and cysteine residues are indicated in bold.

### 8. Expression and purification of recombinant carp CXCa_L1 and carp CXCL8_L2

Vectors pET15new_carpCXCa_L1 and pET15new_carpCXCL8_L2 were used to transform *E. coli* BL21_CodonPlus(DE3)_RIL (Agilent Technologies). A single colony was picked from an overnight plate, grown in LB containing chloramphenicol, ampicillin and 1% glucose. The culture was spread on a LB agar plate and grown overnight at 37°C. Bacteria were harvested with an inoculation loop and resuspended in 10 ml LB and subsequently in 500 ml LB containing ampicillin and chloramphenicol. The culture was grown at 37°C until an optimal density at 600 nm (OD_600_) of 0.6–0.8 was reached. Gene expression was induced by addition of 1mM IPTG and bacteria were incubated for 3 h while shaking. Bacteria were spun down by centrifugation at 17,000×g, 4°C for 15 min (Sorvall Instruments, DuPont, RC5C, GS-3 rotor). The bacterial pellet was resuspended in 40 ml buffer B (20 mM Tris, 500 mM NaCl) containing 0.1 mg/ml lysozyme (Merck). The bacteria suspension was transferred to a 50 ml tube and incubated under rotation for 30 min at RT. Five ml of buffer C (100 mM DTT, 50 mM EDTA, 10% Triton X-100) was added, mixed and stored overnight at −20°C. Subsequently three freeze/thaw cycles were performed to lyse the bacteria. After the last thawing step, 3 ml 0.5 M MgCl_2_ and 75 units benzonase nuclease (Novagen) was added and incubated for 15 min. Total protein lysate was centrifugated at 10,000×g for 15 min at 4°C. The pellet, containing CXCa_L1 or CXCL8_L2 inclusion bodies, was washed in buffer I (50 mM Tris-HCL, 500 mM NaCl) with 1% triton-X100, centrifuged at 10,000×*g* for 10 min at 4°C. Pellet was dissolved in 3 ml buffer II (50 mM Tris-HCL, 500 mM NaCl, 10 M Urea, 15 mM imidazole) and centrifuged at 10000 g for 10 min to remove insoluble material. The 6xHis-tagged protein was purified by immobilized metal affinity chromatography (IMAC) on chelating sepharose fast flow (Amersham-Biosciences) charged with Ni^2+^ according to the manufacterer's protocol. The column was equilibrated with buffer IV (20 mM Tris-HCl, 500 mM HCl, 8 M urea, 25 mM imidazole) and dissolved inclusion bodies were applied to the column. The bound protein was washed with buffer IV (20 mM Tris-HCl, 500 mM HCl, 8 M urea, 25 mM imidazole) containing 1% Triton-X100, followed by buffer IV (20 mM Tris-HCl, 500 mM HCl, 8 M urea, 25 mM imidazole) containing 1% Triton-X114. Triton X-114 was removed by a wash step with buffer VI (20 mM Tris-HCl, 500 mM HCl, 6 M urea) containing 40% (W/V) isopropanol, followed by buffer IV (20 mM Tris-HCl, 500 mM HCl, 8 M urea, 25 mM imidazole) to restore the concentration of urea. Protein was eluted by adding elution buffer (50 mM Tris-HCl, 500 mM HCl, 8 M urea, 250 mM imidazole) and refolded by diluting the eluted fractions 10× in refolding buffer (50 mM Tris-HCl, 0.5 M L-arginine, 0.1 mM oxidized glutathione and 0.5 mM reduced glutathione) and incubation overnight at 4°C. The protein was dialyzed against 1× PBS and centrifuged at 4000 g for 30 min at 4°C to remove any precipitation, filtered with a sterile 0.2 µM filter and stored at −80°C. Protein concentrations were determined by the Micro BCA™ Protein assay kit (Pierce). Protein samples were verified on a Bis-Tris-HCl buffered polyacrylamide gel (NuPAGE, Invitrogen) and protein bands were analyzed by matrix-assisted laser desorption ionization- time of flight (MALDI-TOF) as described earlier [42].

### 9. *In vitro* chemotaxis assay

Chemotactic activity for recombinant carp CXCa_L1 and CXCL8_L2 was analyzed *in vitro* in a 48-well microchemotaxis chamber (Neuro Probe Inc., Maryland, USA) as described earlier. Briefly, lower compartments were filled with either negative controls (serum-free RPMI for random cell migration or recombinant carp IFN-γ-2 (20 ng/ml) (personal observation), or positive control (zymosan-activated carp serum (ZAS); [43]) for the chemotaxis assay. Recombinant carp CXCa_L1 and CXCL8_L2 were tested at 2, 20, 200, 400 and 2000 ng/ml in serum-free RPMI. The lower compartment was covered with a 5 µm pore nitrocellulose filter (Nucleopore membrane, Neuro Probe Inc. Maryland, US) and wells of the upper compartment were loaded with phagocyte suspensions (2×10^6^ cells/ml), prepared as described in paragraph 5.2.1. Chambers were incubated for 3 hours at 27°C and after incubation, filters were fixed and hematoxylin-stained as described earlier [43]. Cells that had migrated into the filter were counted in three high-power fields (400×) using a light microscope the average number of cells per field was determined.

### 10. Statistical analysis

Data were expressed as mean ± standard deviation (SD) and significance of differences was determined using student t-test.

## Results

### 1. Identification of two new putative CXCL8-like genes in zebrafish

**Figure 1:**
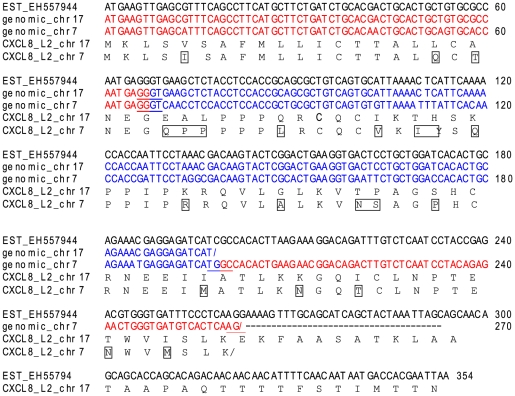
Multiple nucleotide alignment of zebrafish CXCL8 sequences. With zebrafish EST EH557944, zebrafish CXCL8_L2_chr7 (3 exons) and zebrafish CXCL8_L2_chr17 (2 exons). EST EH557944 and CXCL8_L2_chr17 are 100% similar. Alternating exons are presented in red and blue and putative splicing sites are underlined. The translation for both putative genes is presented and differences are boxed.

A second CXCL8-like gene was recently described in carp, that was clearly distinct from the one earlier described in this species (CXCa) and in other fish, indicating that two CXCL8-lineages exist in fish. The first set of CXCL8 like genes in fish, including carp CXCa, will be referred as lineage one (L1) and the new carp CXCL8 and related genes will be referred to as lineage two (L2). We screened the zebrafish genome to identify counterparts for carp CXCL8 of the second lineage (CXCL8_L2). By tBLASTn we retrieved reliable hits on chromosome seven and seventeen, corresponding with partial gene models with three exons (chromosome 7) and two exons (chromosome 17). The new putative CXCL8 genes were named *CXCL8_L2_chr7* and *CXCL8_L2_chr17* and share 89% nucleotide sequence similarity to each other, indicating that they are not duplication artifacts of the genome assembly, but rather distinct genes. No matching EST sequences were retrieved for the gene model located on chromosome seven, but the one from chromosome 17 was 100% similar to four identical ESTs indicating that this gene is expressed. These ESTs contain a full-length CXC ORF including the exons missing on chromosome 17, which could not be retrieved in the genome assembly using BLAT and tBLASTn (Fig 1). The exon/intron structure of *CXCL8_L2_chr7* and *CXCL8_L2_chr17* is rather similar to the one of CXCL8 genes from lineage 1 such as the one present on the zebrafish chromosome 1 (*CXCL8_L1_chr1*) (Fig. S1a). BLASTp search with both gene sequences retrieved mammalian CXCL8 as the most similar CXC sequence. *CXCL8_L2_chr7* and *CXCL8_L2_chr17* share less about 40% protein sequence similarity with zebrafish *CXCL8_L1_Chr1*, corresponding to a distant homology (Fig. S1b-d).

### 2. The second CXCL8 lineage is specific to cyprinids

To search for CXCL8 genes from the second CXCL8 lineage in other fish, we analyzed EST databases and the full genome assembly of tetraodon, fugu, stickleback and medaka. TBLASTn search was performed with CXCL8 protein sequences from both lineages: zebrafish CXCL8_L1_chr1, carp CXCL8_L2, and zebrafish CXCL8_L2_chr17. While tBLASTn and BLASTp analyses identified several sequences belonging to the first CXCL8 lineage with highly significant scores (Table S1) in various fish, no counterpart of CXCL8 from the second lineage could be retrieved outside cyprinids.

An analysis for conserved synteny groups including the three zebrafish CXCL8 genes showed that fish CXCL8_L1 and neighboring genes are part of a synteny block that is well conserved in zebrafish, medaka, tetraodon and fugu. This indicates that fish CXCL8 of the first lineage are true orthologs (Fig. S2a). Genes located in close proximity to zebrafish *CXCL8_L2_chr7* form a conserved synteny group in teleosts, mammals and birds (Fig. S2b), but no CXCL8 or other CXC genes are described on corresponding chromosomes. Genes located in close proximity to CXCL8_L2_chr17 form a conserved synteny group only with mammals and birds (Fig. S2c), which also lacks CXC genes.

**Figure 2:**
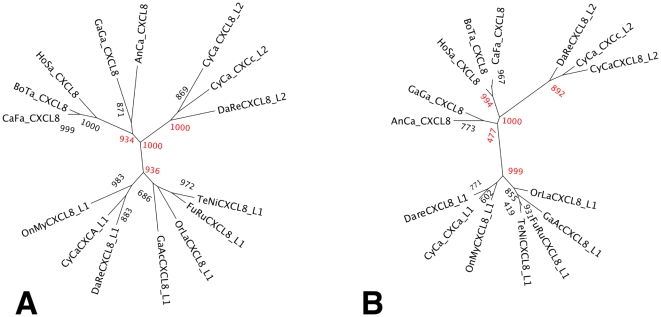
CXCL8 phylogeny. Trees were constructed by Neighbour Joining (**A**) or Maximum Likelyhood (**B**) with complete deletion of gaps from the alignment. Relevant bootstrap (N = 1000) values are indicated in red. CXCL8 accession numbers are as follows: anolis AnCa_CXCL8, ENSACAG00000011382; chicken GaGa_CXCL8, P08317; common carp lineage 1 CyCa_CXCa_L1, CAD13189; common carp lineage 2 CyCaCXCL8_L2 BAH98111; common carp lineage 2 CyCaCXCc_L2 EC394283; cow BoTa_CXCL8, P79255; dog CafaCXCL8, P41324; fugu lineage1 FuRuCXCL8_L1, 49532767; human HoSaCXCL8, P10145; medaka lineage_1 OrLaCXCL8_L1, ENSORLG00000005096; rainbow trout lineage 1 OnMyCXCL8, CAC33585; stickleback lineage 1 GaAcCXCL8_L1, ENSGACG00000001729; tetraodon lineage_1 TeNiCXCL8_L1, ENSTNIG00000017810; zebrafish lineage 1 DaReCXCL8_L1, XP_001342606; zebrafish lineage 2 DaReXCL8_L2, EH441857.

Phylogenetic trees constructed for fish and tetrapod CXCL8 using NJ and ML methods shows two distinct clusters for teleost CXCL8 (Fig. 2). One cluster corresponds with the fish CXCL8_L1 and includes zebrafish CXCL8_L1_chr1 and carp CXCa_L1. The second clusters consists of carp CXCL8_L2, zebrafish CXCL8_L2_chr7 and zebrafish CXCL_L2_chr17. This indicates that two CXCL8-lineages are present in fish, of which the first is conserved among fish and the second is specific for cyprinids. Instability of tree nodes at the split of reptile, mammalian and fish CXCL8_L2 clusters, using NJ or ML as tree construction method, render the respective evolutionary distances of fish CXCL8_L1 and fish CXCL8_L2 to mammalian CXCL8 difficult to assess precisely.

### 3. Constitutive expression of carp CXCL8_L2 gene in immune organs and brain

**Figure 3:**
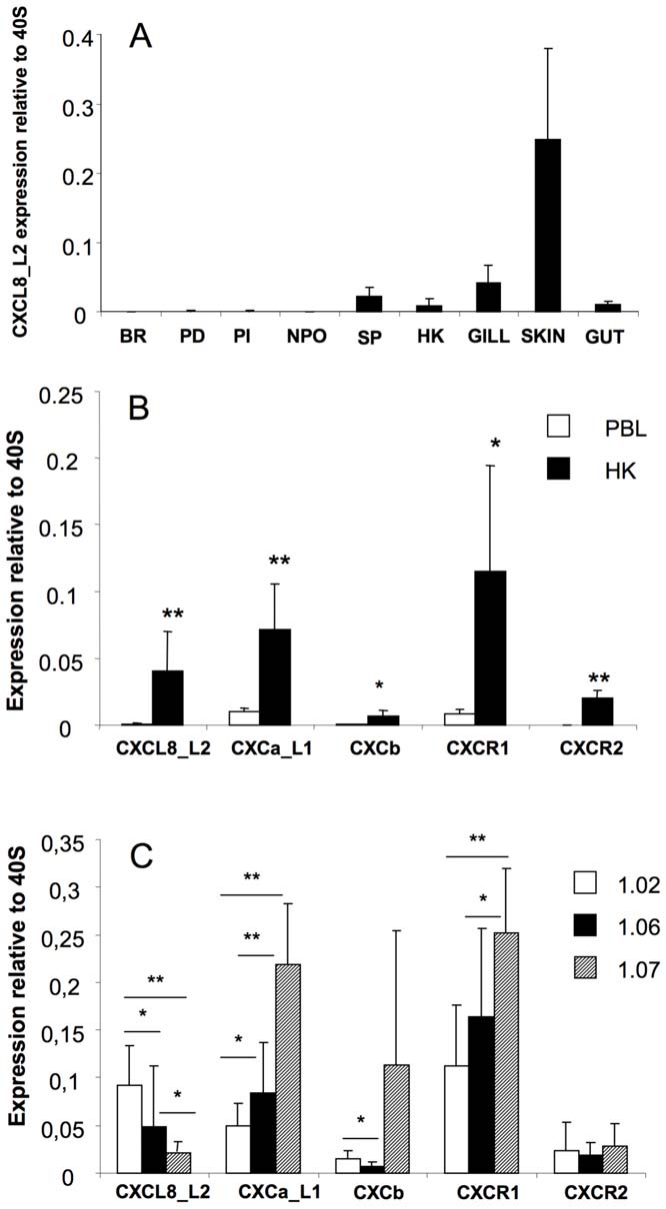
Constitutive gene expression of carp chemokines and chemokine receptors. Constitutive expression of chemokine (CXCL8_L2, CXCa_L1, CXCb) and chemokine receptor (CXCR1 and CXCR2) genes in brain areas and immune-related organs (A), in peripheral blood leukocytes or head kidney phagocytes (B), in monocyte/lymphocyte- (1.020–1.060 g/cm^3^), macrophage- (1.060–1.070 g/cm^3^) and granulocyte- (1.070 to 1.083 g/cm^3^) enriched fractions from head kidney (C). Expression was determined by quantitative real time PCR and plotted relative to the expression of 40S ribosomal protein s11 gene. Bars represent the average expression ± SD in organs or tissues obtained from four individual carp. (n = 4–5 for Fig. 3A and n = 5–9 for Fig. 3B and Fig. 3C). BR, brain, PD, pars distalis; PI, pars intermedia, NPO, nucleus pro-opiticus of hypothalamus; SP, spleen; HK, head kidney, PBL, peripheral blood leukocytes. *, p<0.05, **, p<0.01.

The expression of carp CXCL8_L2 was determined in various organs and tissues. CXCL8_L2 showed high constitutive expression in immune organs (spleen, head kidney). Highest constitutive expression was observed in the periphery, in gills, skin and to a lesser extent gut. In whole brain, a much lower constitutive expression of CXCL8_L2 was observed and also in the nucleus preopticus region of the hypothalamus (NPO), the brain area of specific interest for the stress response, as well as in the pituitary pars distalis (PD) and pars intermedia (PI) the constitutive expression of CXCL8_L2 gene was low (Fig. 3a). Moreover, after prolonged restraint, (24 h netting) no significant changes of expression of the CXCL8_L2 gene were observed, neither in the hypothalamic brain areas, nor in the head kidney (data not shown).

### 4. Constitutive expression of chemokine and chemokine receptor genes in leukocyte populations

Both in HK phagocytes and PBLs, constitutive expression of CXCL8_L2 and CXCa_L1 genes was higher than expression of CXCb. Furthermore the expression of CXCR1 was higher than CXCR2. Expression of all studied genes was higher in HK phagocytes then in PBLs (Fig. 3b).

In density-separated fractions of HK leukocytes, the highest expression of the CXCL8_L2 gene was measured in the monocyte/lymphocyte fraction. Granulocyte enriched fractions showed high basal expression of the genes for CXCa_L1, CXCb and CXCR1 (Fig. 3c). Macrophage enriched fractions showed intermediate levels of expression of CXCL8_L2, CXCa_L1 and CXCR1. Expression of the CXCR2 gene was low in all studied HK fractions.

### 5. Expression of chemokine and chemokine receptor genes after *in vitro* stimulation of carp leukocytes

#### 5.1. Chemokine and chemokine receptor gene expression in stimulated PBLs

**Figure 4:**
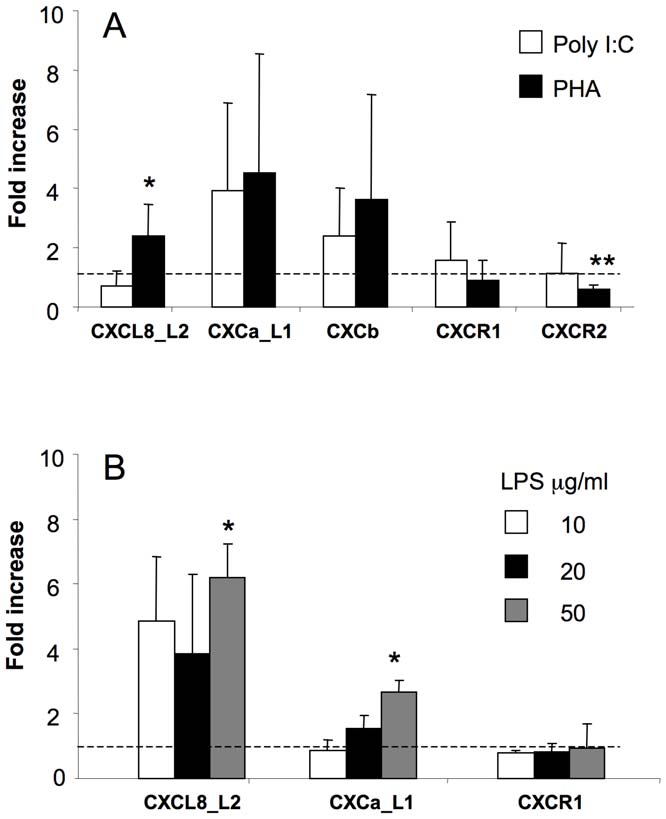
Carp chemokine and chemokine receptor gene expression in stimulated PBLs. Expression of chemokine (CXCL8_L2, CXCa_L1, CXCb) and chemokine receptor (CXCR1 and CXCR2) genes in peripheral blood leukocytes stimulated with poly-inosinic poly cytidiylic (Poly I∶C, 50 µg/ml) or phytohemagglutinin (PHA, 10 µg/ml) (A) or with different concentration of lipopolysaccharide (LPS) Data are shown as x-fold increase of mRNA expression compared to non-stimulated control cells standardized for the housekeeping gene 40S ribosomal protein s11. Averages (n = 4 for Fig. 4A and n = 6 for Fig. 4B) and SD are given. *, p<0.05.

While their constitutive expression is low, chemokines are inducible in PBLs. Significant increase of CXCL8_L2 gene expression was observed 4 h after PHA (10 µg/ml, Fig. 4a), or LPS (50 µg/ml, Fig. 4b), but not Poly I∶C (50 µg/ml, Fig. 4a), stimulation. The same dose of LPS also induced upregulation of CXCa_L1 expression (Fig. 4b). Expression of the CXCR1 gene in PBLs was unchanged upon stimulation (Fig. 4a, b), while expression of CXCR2 was downregulated upon PHA treatment (Fig. 4a). Both CXCb and CXCR2 gene expression was not stimulated upon LPS treatment (data not shown).

#### 5.2. Chemokine and chemokine receptor gene expression in stimulated HK leukocytes

**Figure 5:**
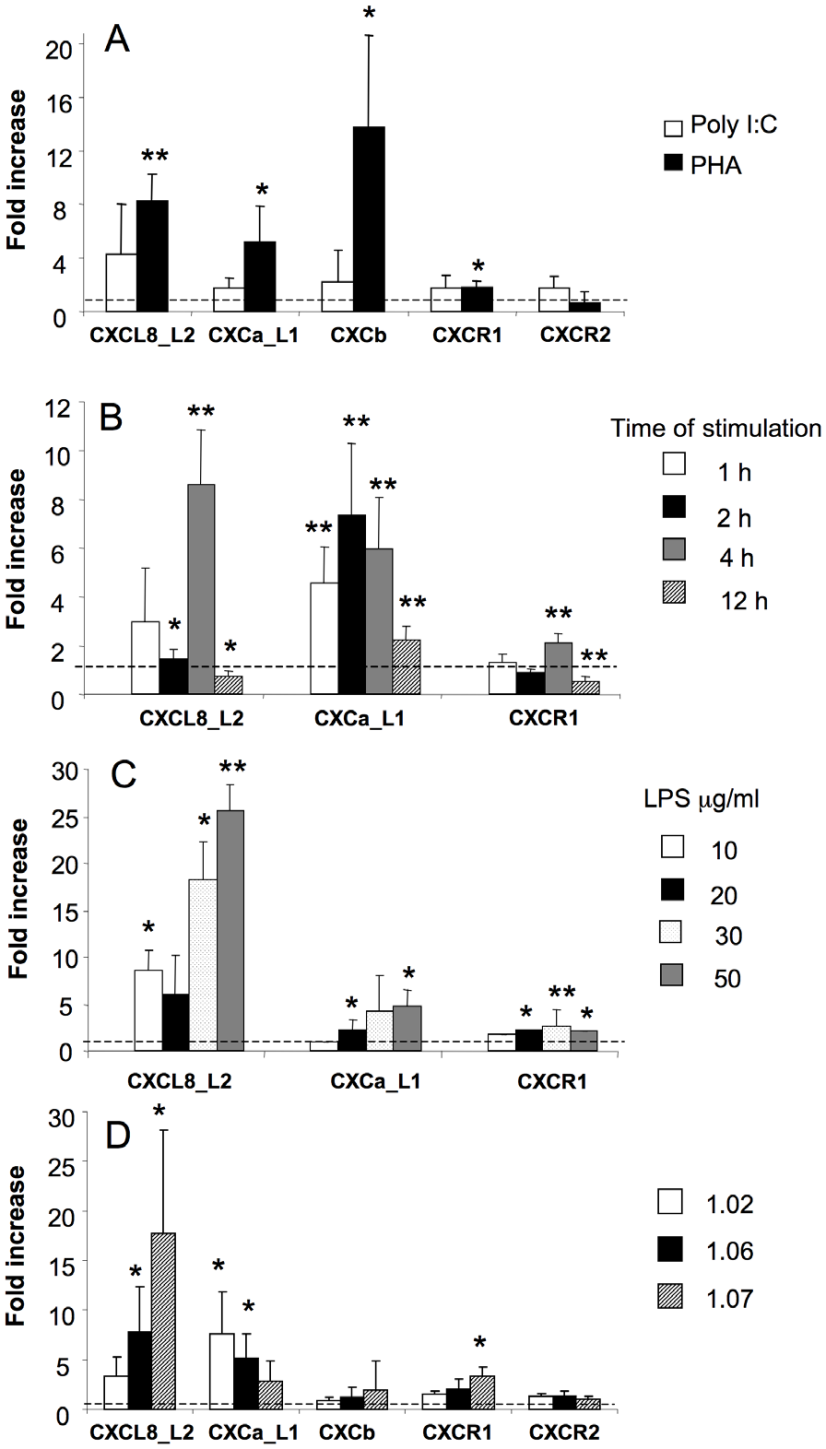
Carp chemokine and chemokine receptor gene expression in stimulated HK leukocytes. Expression of chemokine (CXCL8_L2, CXCa_L1, CXCb) and chemokine receptor (CXCR1 and CXCR2) genes in head kidney phagocytes (A–C) or in monocyte/lymphocyte- (1.020–1.060 g/cm^3^), macrophage- (1.060–1.070 g/cm^3^) and granulocyte- (1.070 to 1.083 g/cm^3^) enriched fractions from head kidney (C). Cells were stimulated with poly-inosinic poly cytidiylic (Poly I∶C, 50 µg/ml) or phytohemagglutinin (PHA, 10 µg/ml) (A) or with lipopolysaccharide (LPS) (B–D). Data are shown as x-fold increase of mRNA expression compared to non-stimulated control cells standardized for the housekeeping gene 40S ribosomal protein s11. Averages (n = 4–6 for Fig. 5A–C and n = 9 for Fig. 5D) and SD are given. *, p<0.05, **, p<0.01.

Significant upregulation of CXCL8_L2, CXCa_L1 and CXCb gene expression was observed in HK phagocytes at 4 h of PHA (10 µg/ml) stimulation. Stimulation levels ranged from 5-fold for CXCa_L1 to 14-fold for CXCb (Fig. 5a). Expression of the CXCL8_L2 gene started to increase in head kidney phagocytes at 2 h reaching a 9-fold increase at 4 h and a subsequent downregulation at 12 h after *in vitro* LPS (50 µg/ml). Gene expression of CXCa_L1 rapidly increased after stimulation (1 h), remained higher till 4 h of stimulation and was less but still significantly increased at 12 h. Also upon LPS treatment, significant increase of expression of the CXCR1 gene was observed in HK phagocytes at 4 h of stimulation, followed by a decrease at 12 h (Fig. 5b). The dose-response relationship for CXCa_L1 and CXCL8_L2 responses to LPS differed. At 4 h after stimulation, doses of 10, 30 and 50 µg/ml of LPS stimulated expression of the CXCL8_L2 gene with a maximum response at 50 µg/ml, whereas CXCa_L1 gene expression was upregulated by doses of 20, 30 and 50 µg/ml of LPS (Fig. 5c). CXCb and CXCR2 expression were not responsive to LPS treatment (data not shown).

Compared to lymphocyte/monocytes fractions and macrophage fractions the expression of CXCL8_L2 in the HK granulocytes could be stimulated with LPS to a much higher extent, both in fold expression and even more in absolute terms. For CXCa_L1 the reverse was found (Fig. 5d). CXCR1 gene expression was upregulated in the HK granulocyte fraction, while all leukocyte subpopulations did not show changes in expression of CXCb and CXCR2 genes upon LPS-stimulation (Fig. 5d).

### 6. *In vivo* expression of chemokine and chemokine receptor genes in peritoneal leukocytes during zymosan-induced peritonitis

**Figure 6:**
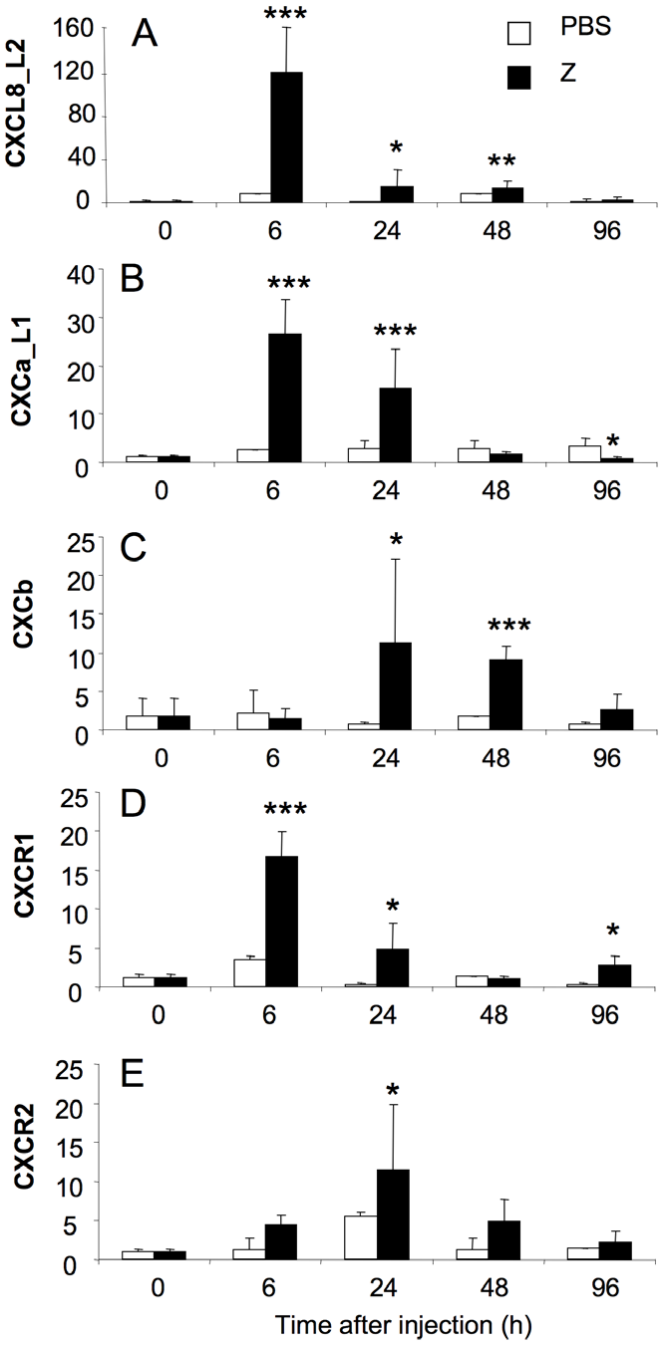
Carp chemokine and chemokine receptor gene expression during zymosan-induced peritonitis. Expression of chemokine (CXCL8_L2, CXCa_L1, CXCb) and chemokine receptor (CXCR1 and CXCR2) genes in peritoneal leukocytes 0, 6, 24, 48, 96 h after zymosan (Z, 2 mg/ml ie 0.5ml/50g b.w.) induced peritonitis. cDNA of n = 4–9 fish was used as template for quantitative real time PCR. Messenger RNA expression is shown as x-fold increase compared to control saline-treated animals at time 0 (PBS) standardized for the housekeeping gene 40S ribosomal protein s11. Averages (n = 4–9, data combined from 3 separate experiments) and SD are given. *, p<0.05, **, p<0.01, ***, p<0.001.

Intraperitoneal injection of zymosan induced acute inflammation in the peritoneal cavity, manifested by massive influx of phagocytes into the focus of inflammation and changes in expression of pro- and anti-inflammatory mediator genes [33]. Expression of the CXCL8_L2 gene was quickly and significantly upregulated in peritoneal leukocytes at 6–48 h after zymosan injection, with a maximal increase at 6 h of inflammation (Fig. 6a). Increased expression of the CXCa_L1 gene was observed at 6 and 24 h of peritonitis, while 96 h after zymosan injection CXCa_L1 gene expression was downregulated (Fig. 6b). A significant increase of expression of the CXCb gene in the peritoneal leukocytes was recorded during later stages, 24 and 48 h after the onset of the inflammatory reaction (Fig. 6c).

Also the level of expression of the CXCR1 gene was upregulated at 6, 24 and 96 h of inflammation with an early peak at 6h (Fig. 6d). Upregulation of expression of the CXCR2 gene was only statistically significant at 24 h after zymosan stimulation (Fig. 6e).

### 7. Expression of chemokine and chemokine receptor genes in gills after hyperosmotic shock

**Figure 7:**
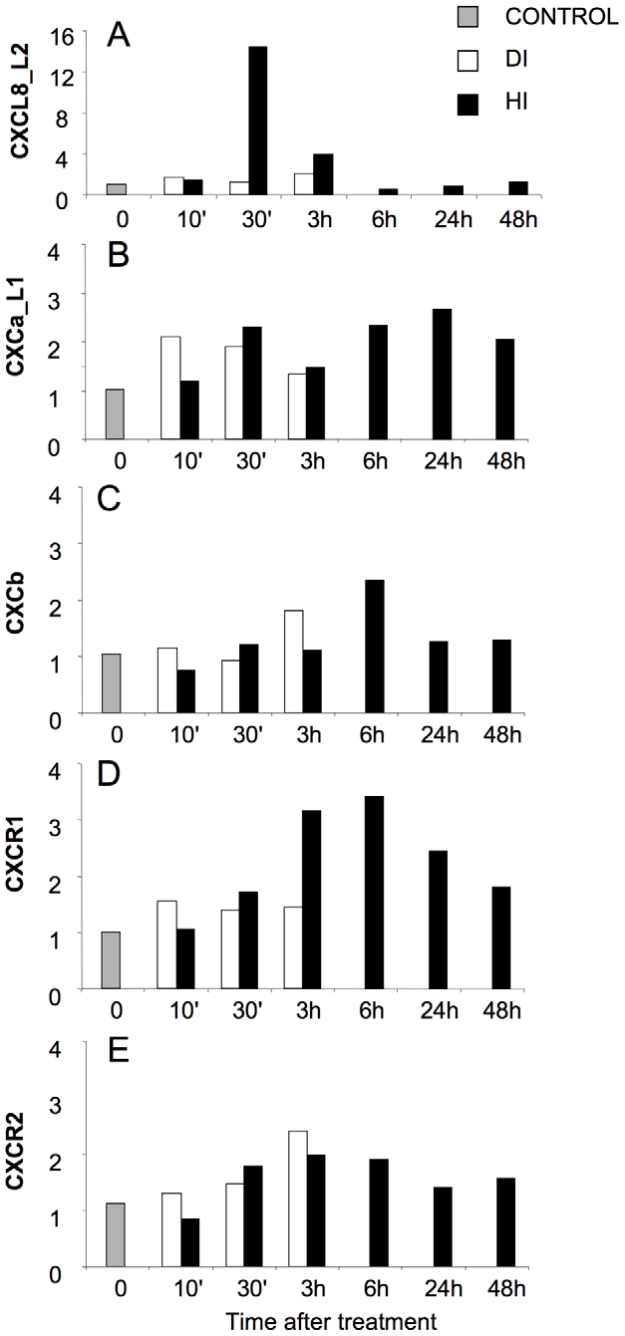
Carp chemokine and chemokine receptor gene expression after hyperosmotic shock. Expression of chemokine (CXCL8_L2, CXCa_L1, CXCb) and chemokine receptor (CXCR1 and CXCR2) genes in gills upon hyperosmotic immersion (HI) or direct immersion (DI) with *A. salmonicida* bacterin-FITC (2.4×10^7^ bacteria/ml). Gene expression is shown at 0, 10 and 30 min and 3, 6, 24, 48 hours after vaccination. Messenger RNA expression is shown as x-fold increase compared to control non-treated animals at time 0 and standardized for the housekeeping gene 40S ribosomal protein s11. Averages (n = 2–3) are given.

Hyperosmotic treatment induced mild disruption of the integrity of the gill epithelia. After immersion vaccination with LPS-DTAF, granulocytes were quickly leaving the head kidney and appeared in PBL. An inflammatory reaction was observed in the gills with an IL-1β and iNOS peak at 3h after immersion vaccination [39]. Gene expression of CXCL8_L2 increased 14-fold in HI-treated fish within 30 min following the immersion vaccination with LPS-DTAF, which decreased to 4 fold after 3 h to finally return to baseline levels within 6 h (Fig. 7a). While expression of CXCR1 was upregulated in the HI-group 3–24 h after treatment (Fig. 7d), only small changes in gene expression of CXCa_L1 (2–3 fold) (Fig. 7b) and CXCb (2 fold) (Fig. 7c,) were detected between HI- and DI- treated animals. Again, expression of CXCR2 remained unaltered (Fig. 7e).

### 8. Recombinant carp CXCa_L1 and CXCL8_L2 are both chemotactic for carp phagocytes

**Figure 8:**
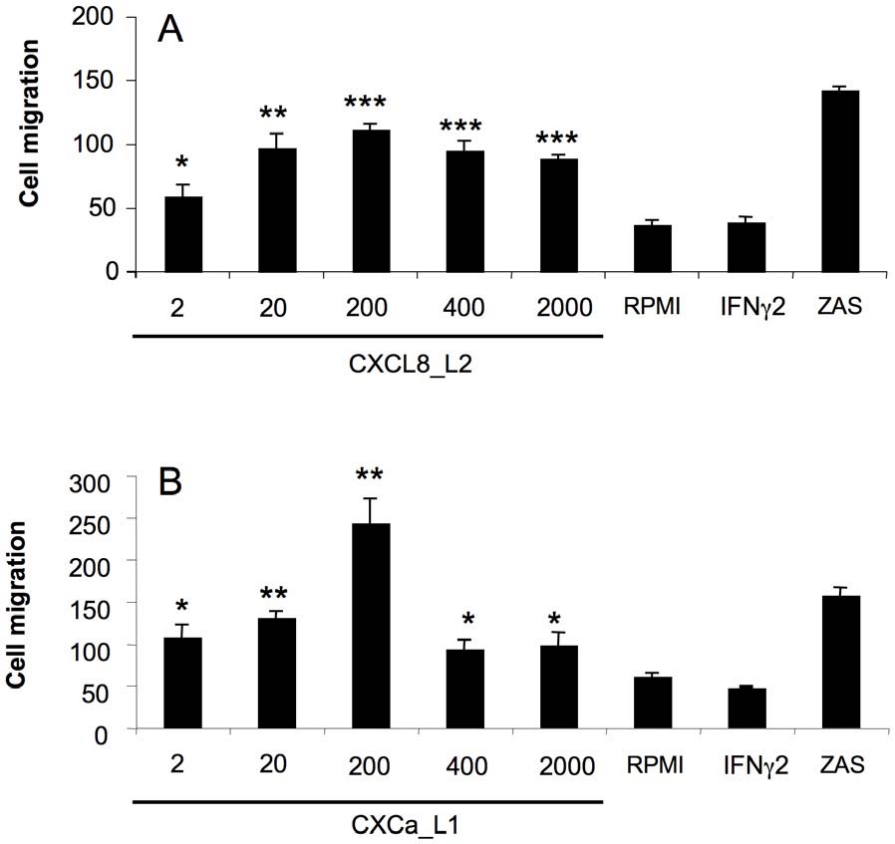
Migration of phagocytes towards recombinant carp CXCa_L1 and CXCL8_L2 chemokines. *In vitro* chemotaxis of carp phagocytes to (**A**) recombinant carp CXCL8_L2 (2; 20; 200; 400; 2000 ng/ml) and (**B**) CXCa_L1 (2; 20; 200; 400; 2000 ng/ml) after 3 hr incubation. Serum-free RPMI and recombinant carp IFN-γ-2 (20 ng/ml) were included as negative controls, zymosan-activated serum (ZAS, non-diluted) as positive control. Cell migration is indicated as average cell number of three fields per well. Averages (n = 3) and SD are indicated. *, p<0.05, **, p<0.01, ***, p<0.001.

To investigate whether CXCL8 chemokines from both lineages are chemotactic, recombinant proteins were prepared for carp CXCa_L1 and carp CXCL8_L2 (Fig. S3). N-terminal 6xHis-tagged recombinant proteins were expressed in *E. coli* and purified on a nickel-column; analysis on NuPAGE gel confirmed protein purity. MALDI-TOF analysis validated protein sequences. Chemotactic activity was assessed *in vitro* with chemotaxis chambers for carp head kidney phagocytes. Compared to RPMI or recombinant IFN-γ-2 prepared in parallel to chemokines, both recombinant carp CXCa_L1 and CXCL8_L2 showed a strong chemotactic activity, with a maximum response matching the one induced by zymosan-activated carp serum, at 200 ng/ml (Fig. 8).

## Discussion

In contrast to mammals, at least two CXCL8-like lineages are present in teleosts. Lineage 1, *CXCL8_L1* consists of chemokine genes that are conserved among multiple fish species and include carp *CXCa_L1* [21] and zebrafish *CXCL8_chr1* [26], as well as *IL-8* of flounder [19], trout [20], haddock [22], fugu [23], atlantic cod [24] black seabream [25] and three striped trumpeter [27]. Sequence similarity, phylogeny and synteny analyses show that fish *CXCL8_L1* genes are orthologues. Although zebrafish *CXCL8_L1_chr1* is located in a synteny group that is partly conserved in mammals (our results, [34]), orthology with mammalian CXCL8 could not be validated by phylogeny, probably due to the low phylogenetic signal of these short sequences subjected to fast evolution. On the other hand, CXCL8 sequences from the second lineage appeared to be slightly closer to tetrapod CXCL8 [35], suggesting that they may represent their true counterpart. Surprisingly, these fish CXCL8_L2 genes, including the recently identified carp CXCL8 [35] and the newly described putative genes in zebrafish on chromosome 7 and 17 (this report) were found only in cyprinids. Since common carp has a tetraploid genome, two or four CXC8_L2 genes may be present, depending on when the duplication event has occurred that has led to the two CXCL8_L2 genes in zebrafish. However, gene loss occurs frequently after duplications, and the determination of the number CXCL8_L2 genes in carp will have to wait the complete genome of this species. Synteny analysis did not provide any clue about their origin. Zebrafish *CXCL8_L2_chr7 and 17* are located in gene clusters that are conserved in other vertebrates. In other vertebrates however these clusters do not contain CXC genes located in the neighborhood or even on the corresponding scaffolds or chromosomes. CXCL8 genes have been identified in species that predate the tetrapod-fish split and include lamprey (LFCA-1, [44]), hagfish (BJ653776) and elasmobranchi (AB063299, [26]). However, it is difficult to establish unambiguous and stable phylogeny for CXC genes, due to their small size and high divergence rate (personal observation, [12], [18], [26]. Multiple species-specific CXCL(8)-lineages have probably arisen, as for example the multiple CXCL8 that have been cloned in catfish, or identified by screening of current sequence databases, and do not show clear phylogenetic clustering ([45], personal observations). Thus, it is not clear whether the duplication leading to the two fish CXCL8-lineages occurred before the divergence of fishes and tetrapods, and the similarity of CXC8_L2 with tetrapod CXCL8 has most probably arisen by convergence.

In this context it is interesting to emphasize that no true CXCL8 orthologue has been found in mouse and rat either. In humans, members of the growth-related gene product (GROα, -β and γ, CXCL1, -2 and -3 resp) family also mediate, but in a lesser extent than CXCL8, neutrophil chemotaxis [46]. Mouse and rat orthologs of the GRO family (mouse: KC/CXCL1, MIP-2/CXCL2 and rat CINC-1-3), are major mediators of neutrophil chemotaxis in these species [47], [48], [49], [50], [51], [52].

To investigate the respective functions of the two CXCL8 lineages identified in cyprinids, we performed an extensive *in vitro* and *in vivo* study to characterize their expression in different tissues and in different conditions of cell stimulation. For this purpose, we focused on the carp model, which allows easier sampling and *ex vivo* functional assays.

First indications that both CXCa_L1 and CXCL8_L2 are involved in immunity are provided by their high constitutive gene expression in gills, skin, gut and in classical fish lymphoid organs like spleen and head kidney ([21], [29], this article). This is in contrast to the non-immune chemokine CXCL12 lineage that is highly expressed in brain tissue. Gills, skin and gut form a first line of defense against pathogens and local CXCa_L1 and CXCL8_L2 expression may be attributed to resident leukocytes in these organs. The head kidney in fish, where heamatopoiesis and leukocyte maturation take place [53], [54], is considered as a functional homolog for human bone marrow. CXCa_L1 and CXCL8_L2 mRNA that we observe in head kidney leukocytes may function as a reservoir for fast protein synthesis after head kidney egress. Head kidney granulocytes express constitutively high levels of CXCa_L1, but lower levels of CXCL8_L2 mRNA. Granulocytes have high constitutive expression of the CXCR1 gene, which presumably allows rapid mobilization of these cells in response to infection. In contrast, the expression of CXCR1 by leukocytes that are already in circulation (PBLs) is low. Carp CXCR1 is a candidate receptor for both CXCL8 proteins, due to high sequence similarity between carp and mammalian CXCR1 [55], but receptor-ligand interaction has not been demonstrated yet experimentally.

The *in vitro* induction of both CXCL8–like genes early after stimulation indicates functional homology to mammalian CXCL8. This observation is further corroborated by the early peaks of CXCa_L1 and CXCL8_L2 expression *in vivo*, within 6 h after the onset of zymosan-induced peritonitis and 30 min after the fast recruitment of granulocytes to the gill tissue after HI-induced damage of epithelial surface of the gills. Yet distinct differences in expression profiles in organs, e.g. a relatively low constitutive expression of CXCL8_L2 compared to CXCL8_L1 in gut [29], or in different cell populations as well as differences in timing could be observed, corresponding to specific sub-functionalization.

While the constitutive expression of CXCa_L1 is higher than that of CXCL8_L2 in neutrophilic granulocytes, only the CXCL8_L2 gene showed a considerable upregulation of expression after LPS stimulation. As we could also observe a differential stimulation with regard to time and dose-response of LPS in the total head kidney phagocyte fractions, we conclude that CXCa_L1 and CXCL8_L2 gene expression is subject to distinct regulatory pathways. The promotor region of mammalian CXCL8 contains binding elements for NF-KB and AP-1 (reviewed by [56]) and expression of the CXCL8 gene is inducible directly by pathogens upon recognition of pathogen associated molecular patterns (PAMPS) by the specific pattern recognition receptors (PRRs) as well as by cytokines and growth factors that are activated after detection of an infectious agent (see for reviews [56]–[57]). Further analyses for enhancer elements in the promotor region of CXCa_L1 and CXCL8_L2 will provide more clues on how different transcription factors regulate the expression of both genes in fish, but remain highly difficult to date since the LPS activation pathway is currently not understood in fish. Neither CXCa_L1 nor CXCL8_L2 are induced *in vitro* by Poly I∶C in PBLs or head kidney phagocytes, in contrast to earlier observations for CXCL8_L1 in other fish species [20], [23], [24], [25].

For two *in vivo* models of inflammation, the zymosan induced peritonitis ([33]) and the HI-induced damage of gill epithelia [39], a massive influx of mainly neutrophilic granulocytes is observed during the early phase of reaction. In accordance to the above mentioned *in vitro* results this correlated to a fast and powerful upregulation of predominantly CXCL8_L2. In both models this coincides with the early peak of expression of the pro-inflammatory cytokine IL-1ß, followed by a peak of expression of the pro-inflammatory mediator iNOS. The observation that *in vivo* the upregulation of CXCL8_L2 and also CXCa_L1 correlate in time with the expression of CXCR1, may support our earlier hypothesis that their chemotactic activity is mediated through this receptor.

In contrast to the early CXCL8-like expression, both *in vitro* and during *in vivo* peritonitis or immunization, the expression of CXCb, which is more closely related but not orthologous to CXCL9-11, appears at later time points. In mammals, IFN-γ, together with LPS, are principal inducers for expression of CXCL9, -10 and -11 in neutrophils, thereby mediating T-lymphocyte recruitment via the CXCR3 (for review [57]). In carp, we earlier identified two IFN-γ genes, IFN-γ-1 and IFN-γ-2, of which IFN-γ-2 expression is significantly induced in PHA-stimulated lymphocytes [58]. Results with recombinant carp IFN-γ-2, have demonstrated that carp CXCb is inducible by IFN-γ-2 and is highly synergistic in combination with LPS, thus indicating that carp CXCb expression is regulated in a similar way as mammalian CXCL9, -10 and -11 [59]. Expression of CXCb during the late phase of inflammation suggests that it recruits cells other than neutrophils. Although two CXCR3 genes have been identified for zebrafish [18], we could not find a carp CXCR3 sequence in available databases (personal observations).

Thus, the CXCL8-like genes appear to be at least partial functional counterparts of the human CXCL8. This is corroborated by the finding that *in vivo* expression is correlated with massive influx of phagocytes to the site of infection [33]. As expression and induction patterns of both CXCL8 genes do not coincide, a sub-functionalization is indicated. Sub-functionalization of duplicated genes of common origin is a classical pattern in teleost fish due their additional genome duplication round(s) [60]. Teleost CXCa_L1 and CXCL8_L2 lack an ELR motif upstream the CXC-motif, a motif that is associated with a functional role in leukocyte chemotaxis in mammals [13], but may not be a prerequisite for chemotaxis of teleost leukocytes [25], [31]. Further characterization of the functional diversity of fish CXCL8s therefore required a direct analysis of their chemotactic activity. For that reason the recombinant CXCL8_L2 and CXCa_L1 proteins were made for which we could subsequently demonstrate a clear chemotactic activity of both chemokines towards phagocytes *in vitro*. We now show that despite the lack of an ELR-motif both carp CXCLa_L1 and CXCL8_L2 are potent chemoattractants for head kidney leukocytes. Both recombinant proteins showed a similar optimum dose response, with a decrease in chemotaxis at higher concentrations, probably due to receptor desensitization, as commonly observed for chemokines [13], [31], [46].

In conclusion, although phylogeny and synteny analysis could not confirm true orthology of carp CXC8_L1 and CXCL8_L2 with mammalian CXCL8, based on chemokine gene expression and their chemotactic activity, we hypothesize that these ligands are functional homologs of mammalian CXCL8. We now showed that apart from the carp chemokine CXCa_L1, the newly identified CXCL8_L2 has a crucial biological role in recruitment of neutrophilic granulocytes during the early phase of inflammation.

## Supporting Information

Table S1 Identified CXCL8_L1 genes in tetraodon, fugu, stickleback and medaka. The location on the genome is indicated, the corresponding accession number or reference of ENSEMBL. E-values correspond with tBLASTn results with zebrafish CXCL8_L1_chr1. Amino acid similarity with zebrafish CXCL8_L1_chr1 is indicated.
(0.03 MB DOC)

Figure S1 Zebrafish CXCL8 gene structure and similarity with carp CXCL8. A Schematic representation of intron/exon organization of zebrafish CXCL8 genes on chromosome 1, 7 and 17. Location on the genome of first and last nucleotides for each exon are indicated on top of each gene, intron and exon sizes are indicated under each gene structure. B Similarity in nucleotides between zebrafish CXCL8 genes located on chromosome 1, 7 and 17, determined by ClustalW. C Similarity in amino acids between carp (Cyca, Cyprinus carpio) and zebrafish (Dare, Danio rerio) CXCL8 sequences, determined by ClustalW. D Multiple protein sequence alignment with zebrafish EST_EH557944 (L2), zebrafish CXCL8_L2_chr17, zebrafish CXCL8_L2_chr7 and carp CXCL8_L2. Differences in amino acids in comparison to zebrafish EST_EH557944 are indicated in red.
(0.18 MB PDF)

Figure S2 Syntenic organization of zebrafish CXCL8 genes. Zebrafish CXCL8_L1_chr1 (A), CXCL8_L2_chr7 (B) and CXCL8_L2_chr17 (C). Each reference gene is indicated by a blue arrow and boxed in blue, CXCL8 genes are indicated by a red arrow and boxed in red. Genes that are in synteny are indicated at the left side of the alignment with zebrafish as reference species.
(0.26 MB PDF)

Figure S3 Production of recombinant carp CXCa_L1 and carp CXCL8_L2 in E.coli. Samples were collected at several steps during the preparation and analyzed on polyacrylamide gel.
(1.06 MB PDF)
