# Supplementary material for: CXCL8 Chemokines in Teleost Fish: Two Lineages with Distinct Expression Profiles during Early Phases of Inflammation
Source: PLoS One. 2010 Aug 26;5(8):e12384. doi: 10.1371/journal.pone.0012384 (PMC2928728; doi:10.1371/journal.pone.0012384)
Supplement: Table S1 — Identified CXCL8_L1 genes in tetraodon, fugu, stickleback and medaka. The location on the genome is indicated, the corresponding accession number or reference of ENSEMBL. E-values correspond with tBLASTn results with zebrafish CXCL8_L1_chr1. Amino acid similarity with zebrafish CXCL8_L1_chr1 is indicated. (0.03 MB DOC) [file pone.0012384.s001.doc]

| **Species** | **Location on genome** | **Gene, transcript or protein ID** | **E-value** | **AA Similarity %** |
| --- | --- | --- | --- | --- |
| tetraodon | Chr 18:579,804-580,152 | ENSTNIG00000017810  ENSTNIT00000021199  ENSTNIP00000020966 | 3.7e-17 | 58% |
| Fugu | scaffold 333_210,419-210,733 | AB125645.1  NP_001027759 | 2,0e-26 | 57% |
| Stickleback | Scaffold 882_2783 to 882_3004 | ENSGACG00000001729  ENSGACT00000002257  ENSGACP00000002251 | 2.9e-09; 4.8e-11 | 50% |
| Medaka | Chr1:16,461,557-16,461,441 | ENSORLG00000005096  ENSORLT00000006430  ENSORLP00000006429 | 2.2e-21 | 50% |

**Table S1 Identified CXCL8_L1 genes in tetraodon, fugu, stickleback and medaka.** The location on the genome is indicated, the corresponding accession number or reference of ENSEMBL. E-values correspond with tBLASTn results with zebrafish CXCL8_L1_chr1. Amino acid similarity with zebrafish CXCL8_L1_chr1 is indicated.
