# Supplementary material for: CXCL8 Chemokines in Teleost Fish: Two Lineages with Distinct Expression Profiles during Early Phases of Inflammation
Source: PLoS One. 2010 Aug 26;5(8):e12384. doi: 10.1371/journal.pone.0012384 (PMC2928728; doi:10.1371/journal.pone.0012384)
Supplement: Figure S1 — Zebrafish CXCL8 gene structure and similarity with carp CXCL8. A Schematic representation of intron/exon organization of zebrafish CXCL8 genes on chromosome 1, 7 and 17. Location on the genome of first and last nucleotides for each exon are indicated on top of each gene, intron and exon sizes are indicated under each gene structure. B Similarity in nucleotides between zebrafish CXCL8 genes located on chromosome 1, 7 and 17, determined by ClustalW. C Similarity in amino acids between carp (Cyca, Cyprinus carpio) and zebrafish (Dare, Danio rerio) CXCL8 sequences, determined by ClustalW. D Multiple protein sequence alignment with zebrafish EST_EH557944 (L2), zebrafish CXCL8_L2_chr17, zebrafish CXCL8_L2_chr7 and carp CXCL8_L2. Differences in amino acids in comparison to zebrafish EST_EH557944 are indicated in red. (0.18 MB PDF) [file pone.0012384.s002.pdf]

A

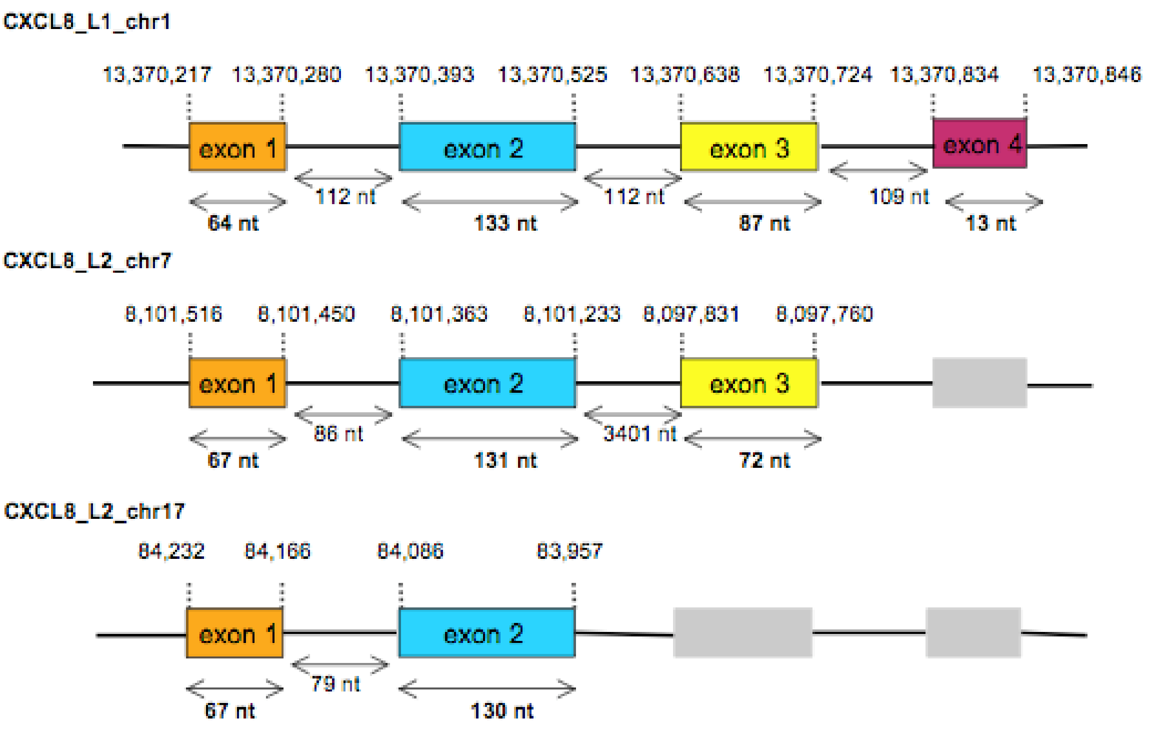

B

SeqA	Name	Len(nt)	Name	Len(nt)	Score
1	CXCL8_L1_Chr1	298	CXCL8_L2_Chr7	270	15
1	CXCL8_L1_Chr1	298	CXCL8_L2_Chr17	197	41
2	CXCL8_L2_Chr7	270	CXCL8_L2_Chr17	197	89

C

SeqA	Name	Len(aa)	Name	Len(aa)	Score
1	Dare_CXCL8_chr1_L1	98	Dare_CXCL8_chr7_L2	118	23
1	Dare_CXCL8_chr1_L1	98	Dare_ESTEH557944/CXCL8_Chr17_L2	118	23
1	Dare_CXCL8_chr1_L1	98	Cyca_CXCa_L1	98	71
1	Dare_CXCL8_chr1_L1	98	Cyca_CXCL8_L2	99	22
2	Dare_CXCL8_chr7_L2	118	Dare_ESTEH557944/CXCL8_Chr17_L2	118	100
2	Dare_CXCL8_chr7_L2	118	Cyca_CXCa_L1	98	30
2	Dare_CXCL8_chr7_L2	118	Cyca_CXCL8_L2	99	54
3	Dare_ESTEH557944/CXCL8_Chr17_L2	118	Cyca_CXCa_L1	98	30
3	Dare_ESTEH557944/CXCL8_Chr17_L2	118	Cyca_CXCL8_L2	99	54
4	Cyca_CXCa_L1	98	Cyca_CXCL8_L2	99	24

D

```
EST_EH557944     MKLSVSAFMLLICTTALLCANEGEALPPPQRCQCIKTHSKPPIPKRQVLGLKVTPAGSHC 60
CXCL8_chr17      MKLSVSAFMLLICTTALLCANEGEALPPPQRCQCIKTHSKPPIPKRQVLGLKVTPAGSHC 60
CXCL8_chr7       MKLSISAFMLLICTTALQCTNEGQPPPPPLRCQCVKIYSQPPIPRRQVLALKVN-SGPHC 59
Carp_CXCL8       MKLTVSAFMLLICTAALLSTTEGRPKSQQLSCRCPRMHSEPAIPANKVLSLRVIPAGPIC 60
                 ***::*********:** .:.**.. .    *:* : :*:*.** .:**.*:*  :*. *

EST_EH557944     RNEEIIATLKKGQICLNPTETWVISLKEKFAASATKLAATAAPAQTTTTFSTIMTTN 117
CXCL8_chr17      RNEEI---------------------------------------------------- 65
CXCL8_chr7       RNEEIMATLKNGQTCLNPTENWVMSLKTQV-------------------------- 89
Carp_CXCL8       KNENIIATMKKGQVCLDPTKDWVISLNEEIKKRNLKSQP----------------- 99
                 :**:*
```

Figure S1 Zebrafish CXCL8 gene structure and similarity with carp CXCL8.
A Schematic representation of intron/exon organization of zebrafish CXCL8 genes on chromosome 1, 7 and 17. Location on the genome of first and last nucleotides for each exon are indicated on top of each gene, intron and exon sizes are indicated under each gene structure. **B** Similarity in nucleotides between zebrafish CXCL8 genes located on chromosome 1, 7 and 17, determined by ClustalW. **C** Similarity in amino acids between carp (Cyca, *Cyprinus carpio*) and zebrafish (Dare, *Danio rerio*) CXCL8 sequences, determined by ClustalW. **D** Multiple protein sequence alignment with zebrafish EST_EH557944 (L2), zebrafish CXCL8_L2_chr17, zebrafish CXCL8_L2_chr7 and carp CXCL8_L2. Differences in amino acids in comparison to zebrafish EST_EH557944 are indicated in red
