# Supplementary material for: CXCL8 Chemokines in Teleost Fish: Two Lineages with Distinct Expression Profiles during Early Phases of Inflammation
Source: PLoS One. 2010 Aug 26;5(8):e12384. doi: 10.1371/journal.pone.0012384 (PMC2928728; doi:10.1371/journal.pone.0012384)
Supplement: Figure S2 — Syntenic organization of zebrafish CXCL8 genes. Zebrafish CXCL8_L1_chr1 (A), CXCL8_L2_chr7 (B) and CXCL8_L2_chr17 (C). Each reference gene is indicated by a blue arrow and boxed in blue, CXCL8 genes are indicated by a red arrow and boxed in red. Genes that are in synteny are indicated at the left side of the alignment with zebrafish as reference species. (0.26 MB PDF) [file pone.0012384.s003.pdf]

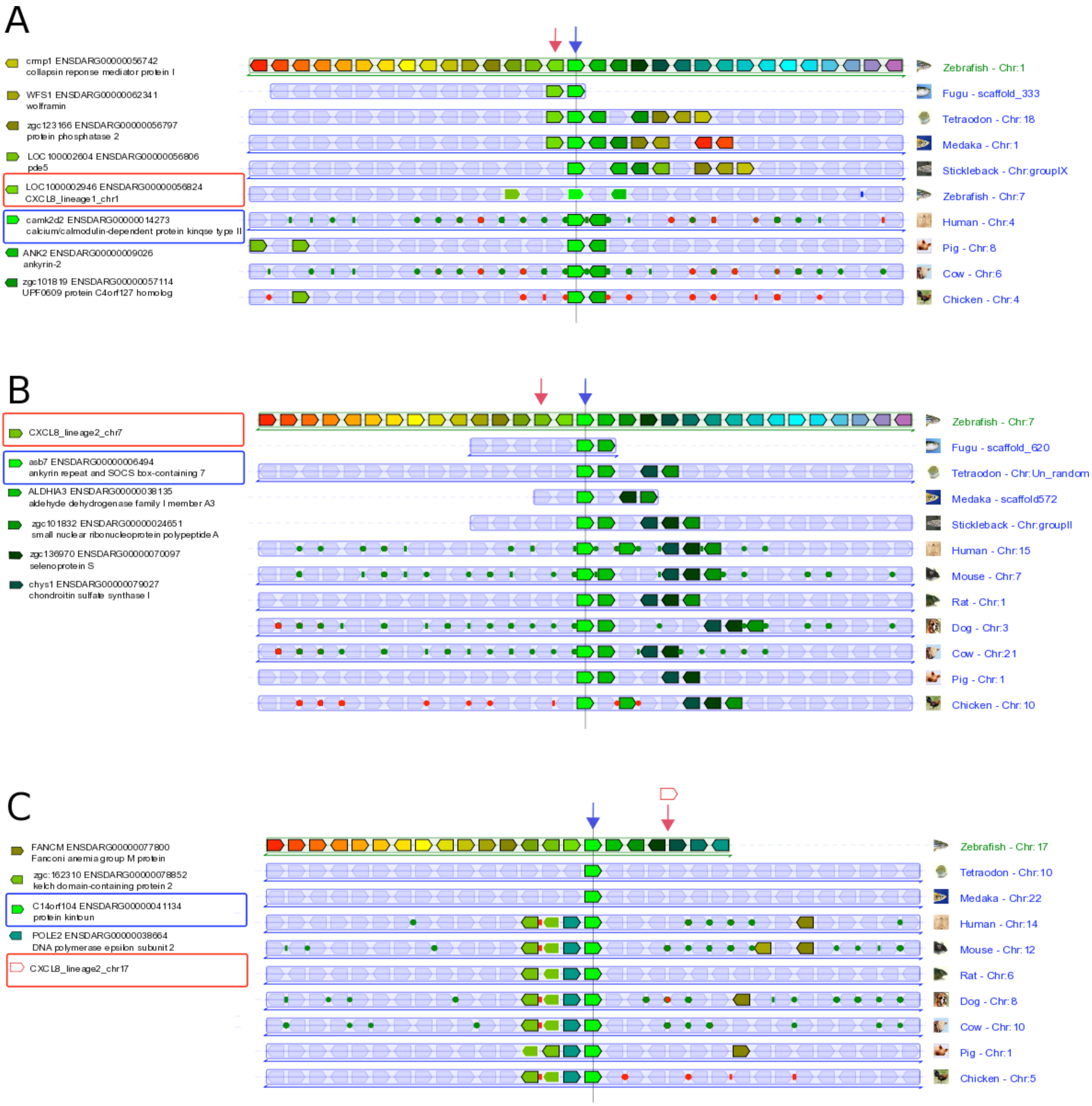

Figure S2 Syntenic organization of zebrafish CXCL8 genes.
Zebrafish *CXCL8_L1_chr1* (**A**), *CXCL8_L2_chr7* (**B**) and *CXCL8_L2_chr17* (**C**). Each reference gene is indicated by a blue arrow and boxed in blue, CXCL8 genes are indicated by a red arrow and boxed in red. Genes that are in synteny are indicated at the left side of the alignment with zebrafish as reference species.
