# Supplementary material for: CXCL8 Chemokines in Teleost Fish: Two Lineages with Distinct Expression Profiles during Early Phases of Inflammation
Source: PLoS One. 2010 Aug 26;5(8):e12384. doi: 10.1371/journal.pone.0012384 (PMC2928728; doi:10.1371/journal.pone.0012384)
Supplement: Figure S3 — Production of recombinant carp CXCa_L1 and carp CXCL8_L2 in E.coli. Samples were collected at several steps during the preparation and analyzed on polyacrylamide gel. (1.06 MB PDF) [file pone.0012384.s004.pdf]

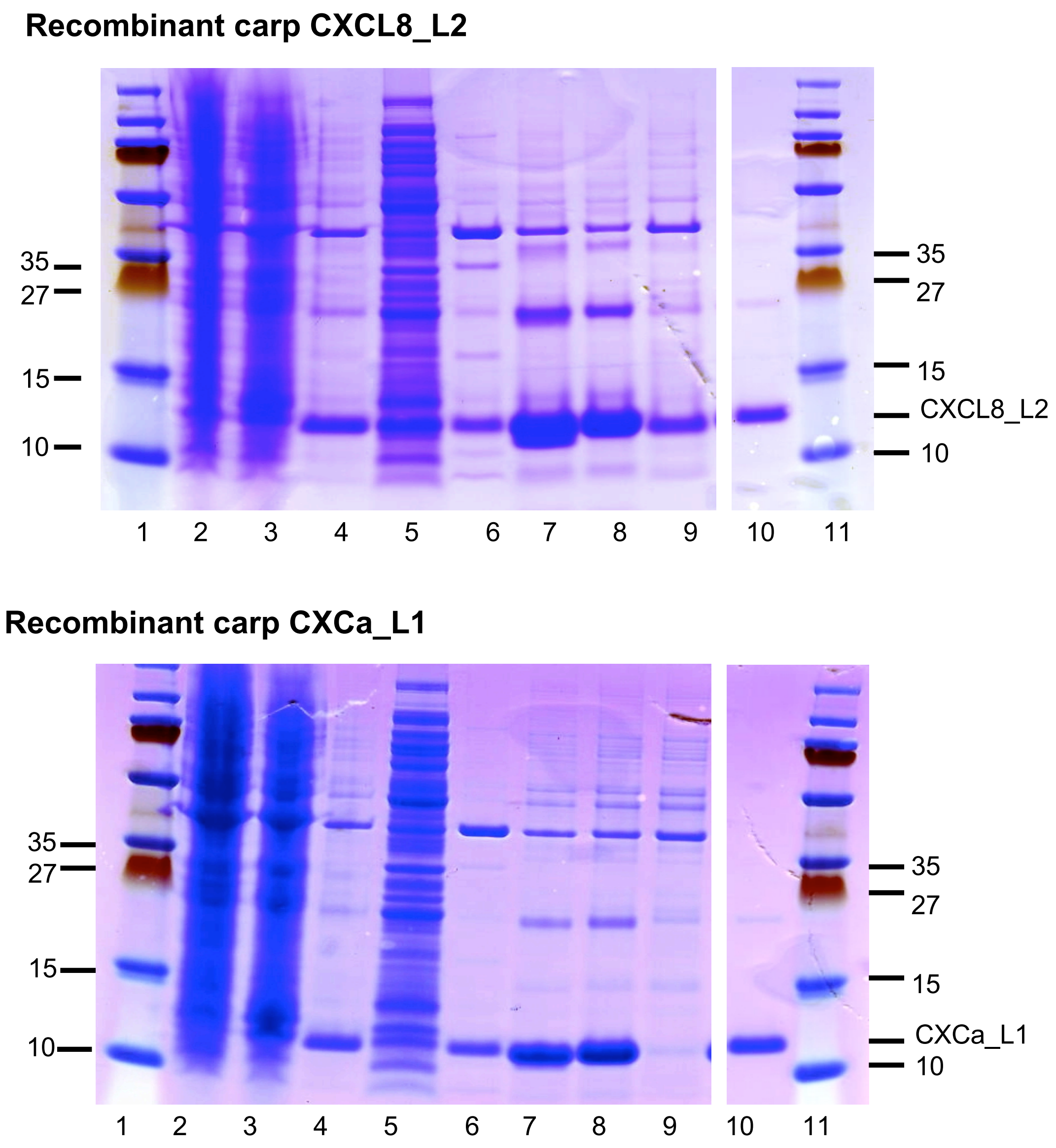

1 = Dual color protein marker
2 = total bacterial lysate non-induced
3 = total bacterial lysate IPTG-induced
4 = non-soluble fraction
5 = soluble fraction
6 = non-resuspended inclusion bodies
7 = inclusion bodies resuspended in urea (total)
8 = inclusion bodies resuspended in urea (supernatant, used on column)
9 = flow-through
10 = eluted protein after dialysis and filtration
11 = dual protein marker

Figure S3 Production of recombinant carp CXCa_L1 and carp CXCL8_L2 in *E. coli*. Samples were collected at several steps during the preparation and analyzed on polyacrylamide gel.
